# *Lactobacillus paracasei* CNCM I-5220-derived postbiotic protects from the leaky-gut

**DOI:** 10.3389/fmicb.2023.1157164

**Published:** 2023-03-20

**Authors:** Francesca Algieri, Nina Tanaskovic, Cindy Cardenas Rincon, Elisabetta Notario, Daniele Braga, Graziano Pesole, Roberto Rusconi, Giuseppe Penna, Maria Rescigno

**Affiliations:** ^1^Postbiotica S.r.l., Milan, Italy; ^2^IRCCS Humanitas Research Hospital, Rozzano, Italy; ^3^Department of Bioscience, Biotechnologies and Environment – DBBA, University of Bari Aldo Moro, Bari, Italy; ^4^Institute of Biomembranes, Bioenergetics and Molecular Biotechnologies, Consiglio Nazionale delle Ricerche, Bari, Italy; ^5^Department of Biomedical Sciences, Humanitas University, Pieve Emanuele, Italy

**Keywords:** postbiotic, leaky-gut, *Lactobacillus paracasei*, tight junction, epithelial barrier integrity, microbiota, gut vascular barrier, *Salmonella typhimurium*

## Abstract

The maintenance of intestinal barrier function is essential for preventing different pathologies, such as the leaky gut syndrome (LGS), which is characterized by the passage of harmful agents, like bacteria, toxins, and viruses, into the bloodstream. Intestinal barrier integrity is controlled by several players, including the gut microbiota. Various molecules, called postbiotics, are released during the natural metabolic activity of the microbiota. Postbiotics can regulate host–microbe interactions, epithelial homeostasis, and have overall benefits for our health. In this work, we used *in vitro* and *in vivo* systems to demonstrate the role of *Lactobacillus paracasei* CNCM I-5220-derived postbiotic (LP-PBF) in preserving intestinal barrier integrity. We demonstrated *in vitro* that LP-PBF restored the morphology of tight junctions (TJs) that were altered upon *Salmonella typhimurium* exposure. *In vivo*, LP-PBF protected the gut vascular barrier and blocked *S. typhimurium* dissemination into the bloodstream. Interestingly, we found that LP-PBF interacts not only with the host cells, but also directly with *S. typhimurium* blocking its biofilm formation, partially due to the presence of biosurfactants. This study highlights that LP-PBF is beneficial in maintaining gut homeostasis due to the synergistic effect of its different components. These results suggest that LP-PBF could be utilized in managing several pathologies displaying an impaired intestinal barrier function.

## Introduction

The intestinal epithelial barrier (IEB) provides the first line of defense for the gastrointestinal tract by separating the body from the external environment. Thus, the integrity of the IEB is important for maintaining homeostasis by mediating the crosstalk between commensal gut microbes and the host as well as preserving the function of intestinal epithelial cells (IECs; Stolfi et al., 2022). IECs form a monolayer that line the IEB and are connected to each other via intercellular junctions, called tight junctions (TJs). TJs create fusion points between epithelial cells which regulate diffusion by forming semipermeable cellular barriers that control paracellular transport to maintain homeostasis. Several types of proteins are involved in TJ formation, such as integral membrane proteins (claudin family proteins), junctional complex proteins [Zonula occludens (ZO)-1, ZO-2, and ZO-3], and cell cytoskeleton structures (microtubules and microfilaments) which together regulate IEB integrity (Camilleri et al., 2012). Indeed, mucosal inflammation can induce modifications in TJ structure resulting in increased permeability of the IEB (Lechuga and Ivanov, 2017). It has been observed that alterations of the IEB structure may lead to pathogen invasion and mucosal dysbiosis (Gitter et al., 2001; Mankertz and Schulzke, 2007; Groschwitz and Hogan, 2009; Martini et al., 2017). This condition, also called the leaky-gut syndrome (LGS), is common to several human diseases, including intestinal, metabolic, and neurological disorders among others (Gitter et al., 2001; Mankertz and Schulzke, 2007; Groschwitz and Hogan, 2009; Martini et al., 2017; Chelakkot et al., 2018; Obrenovich, 2018; Gasaly et al., 2021; Carloni and Rescigno, 2022). Preserving the IEB is thus fundamental to prevent or retard the development of these very debilitating disorders.

Moreover, it is well known that the human body is host to a community of microorganisms, collectively called the microbiota. Taking into account that bacteria outnumber human cells by a 1.3 ratio (Sender et al., 2016), it is important that this coexistence is fine-tuned and led by a symbiotic relationship. However, the gut is a dynamic habitat that is constantly changing based on diet, lifestyle, hygiene, or use of antibiotics, all of which can rapidly modify the microbiota composition and can perturb the intestinal homeostasis (Sommer and Backhed, 2013). Indeed, alterations in IEB permeability (leaky gut syndrome) can also lead to pathogen invasion (e.g., *S. typhimurium* infection) (Spadoni et al., 2015) and mucosal dysbiosis. This can trigger pathological conditions such as inflammatory bowel disease (IBD), celiac disease, irritable bowel syndrome, colorectal cancer, type 2 diabetes, obesity, and many others (Gitter et al., 2001; Mankertz and Schulzke, 2007; Groschwitz and Hogan, 2009; Martini et al., 2017; Gasaly et al., 2021), highlighting the need for a novel strategy to prevent or reduce IEB damage. Moreover, the host microbiota is essential for its ability to enhance food absorption and digestion, modulate the immune system, reduce pathogen growth, and maintain IEB integrity, all of which have a significant influence on the health and physiology of the human being (Hou et al., 2022). The use of probiotics for the treatment of these conditions has also been examined; however, the results are not always conclusive. The therapeutic effects of probiotics are highly dependent on the viability of the bacteria that reach the intestine, suggesting that the metabolic activity of live bacteria is crucial for their functions (Zagato et al., 2014). It is known that the microorganisms in our gut can release different compounds during their natural metabolic activity, known as postbiotics, that can have beneficial effects at the systemic level. The term “postbiotic” refers to any molecule or compound released during the probiotic fermentation process or any secreted metabolite capable of conferring a beneficial effect to the host in either direct or indirect way (Tsilingiri and Rescigno, 2013; Zagato et al., 2020; Aguilar-Toala et al., 2021). Indeed, given the morphology of the gut and the presence of a thick inner mucus layer that protects host cells from a potentially dangerous interaction with the microbiota (Hansson and Johansson, 2010), most of the beneficial activities of the microbiota are associated with their postbiotics.

The aim of the present study was to evaluate the role of *L. paracasei* CNCM I-5220-derived postbiotic (hereafter called LP-PBF) in preserving the integrity of the IEB in different models of *S. typhimurium* infection. Due to the immunomodulatory properties and low toxicity of postbiotics, they could be implemented as a novel approach for re-establishing host-microbe homeostasis and contrast the leaky-gut.

## Materials and methods

### Study workflow

In this work, we performed different *in vitro* and *in vivo* approaches in order to address the role of LP-PBF postbiotic in the maintenance of IEB integrity (Supplementary Figure S1). For the *in vitro* part, we utilized the human epithelial colorectal cancer cell line, Caco-2. This was done through the measurement of the trans-epithelial electrical resistance (TEER), where we studied the integrity of TJs upon *S. typhimurium* infection. Then, using an immunofluorescence assay we assessed the morphology of the TJ protein ZO-1. Both were measured upon pretreatment with LP-PBF or by using LP-PBF-treated *S. typhimurium.* Further, we assessed the integrity of the IEB *in vivo* using a mouse model of *S. typhimurium* infection, where we examined if pretreatment of mice with LP-PBF can preserve the disruption of the IEB upon *S. typhimurium* exposure. This was also carried out by utilizing an LP-PBF pretreated *S. typhimurium* in order to examine if the LP-PBF could interact with both cells and bacteria. Finally, we assessed if the LP-PBF has an anti-biofilm effect on the *S. typhimurium in vitro*.

### Postbiotic production

Postbiotic (short chain Fructooligosaccharides [scFOS] fermented by *L. paracasei* strain CNCM I-5220) LP-PBF was produced by Postbiotica S.r.l. utilizing the innovative PBTech® technology (patent number: WO 2019/149941 A1). Briefly, an inoculum of *L. paracasei* CNCM I-5220 was grown at 37°C in fermentation medium. Next, the collected bacterial biomass is resuspended in a fermentation medium containing scFOS for an additional 24 h. The bacteria were separated by centrifugation (4,000 rpm at 4°C) and supernatant collected. The supernatant was then pasteurized at 90°C for 10 min (to remove any potential live bacteria), supplemented with maltodextrin and powdered by spray drying.

### Bacteria

*Salmonella enterica* serovar typhimurium SL1344 strain FB62 was provided by G. Dougan (The Wellcome Trust Sanger Institute, United Kingdom) and grown in Luria-Bertani (LB) broth. *Salmonella typhimurium* strain SL3261AT, grown at 37°C in LB broth, is an aroA-metabolically defective strain on SL1344 background characterized by an attenuated ability to replicate *in vivo* (Avogadri et al., 2005).

In order to obtain postbiotic-treated *S. typhimurium*, hereafter LP-PBF-treated Sal, bacteria at OD_600_ of 0.6, were incubated for 1.5 h at 4°C with 5 mg/ml of LP-PBF or vehicle, washed and resuspended in LB broth, and was used for either *in vitro* and *in vivo* studies.

### Agar disk diffusion method

Agar plates were inoculated with 6 × 10^7^ CFU of *S. typhimurium* SL1344. Filter paper discs (about 6 mm in diameter), containing 5 mg/ml of LP-PBF, 5 mg/ml of vehicle, or Gentamycin 100 ng/ml (positive control), were placed on the agar surface. The Petri dishes were then incubated at 37°C for 24 h in aerobic conditions and colonies were counted.

### *In vitro* barrier assessment

Caco-2 cells (human epithelial colorectal adenocarcinoma cells) were maintained in DMEM supplemented with 10% Fetal Bovine Serum, 1% Glutamine, 1% Penicillin–Streptomycin.

Experiments were performed by seeding 6 × 10^4^ cells/well on polycarbonate membranes (Transwell 6.5 mm in diameter, 0.4 μm pore size) (Costar Corp). Cell growth was monitored by measuring TEER until confluence by chopstick electrodes (EVOM3, WPI). Upon reaching confluence, cells were pretreated overnight with 5 mg/ml of LP-PBF or the relative control containing Maltodextrins and FOS. The next day, cells were stimulated with *S. typhimurium* SL1344 (25×10^6^ CFU/well) for 1.5 h. Then, *S. typhimurium* SL1344 was removed, and the LP-PBF and controls added for a 4-h recovery phase.

For evaluation of TJs, 150,000 cells/well were seeded in Permanox chambers and allowed to grow for 24 h to form a confluent monolayer before treatment with LP-PBF or control. For immunofluorescent staining, cells were fixed with 4% paraformaldehyde for 10 min and then permeabilized with 0.25% Triton X-100 for 10 min. Staining was performed with 1.25 μg/ml of primary anti-ZO-1-Alexafluor 488 mouse monoclonal antibody (ZO1-1A12, Invitrogen). Before imaging, nuclei were counterstained with 4′,6-diamidin-2-fenilindolo (DAPI). Confocal images were acquired with Leica TCS SP8 laser confocal scanner mounted on Leica DMI 6000B inverted microscope equipped with motorized stage. Junction tortuosity was calculated as a ratio between junction length and Euclidean distance between its ends, as previously published (Thaiss et al., 2018).

### *In vivo S. typhimurium* infection

C57BL/6J mice were purchased from Charles River laboratories France. All mice were maintained in microisolator cages in a specific pathogen-free animal facility. All experiments were performed in accordance with the guidelines established in the 5 Principles of Laboratory Animal Care (directive 86/609/EEC) and approved by the Italian Ministry of Health (927/2022 and 1054/2015).

Mice were treated orally with 135 mg/kg/day of LP-PBF for a period of 10 days (*n* = 8 per group). Control mice received the vehicle control containing maltodextrins and FOS. After pretreatment, mice were infected with 10^9^ CFU of *S. typhimurium* strain SL3261AT *via* oral gavage and after 6 h they were euthanized. Colons were aseptically removed and incubated 30 min at 37°C with gentamycin in order to kill external bacteria. Then, colons were digested with 1 mg/ml Collagenase D (Roche) for 30 min at 37°C. Cells isolated from the colons were lysed with 0.5% sodium-deoxycholate and plated on Columbia agar with sheep blood (Oxoid) to evaluate bacterial dissemination after overnight culture. The livers were also removed aseptically, smashed and filtered on a 70 μm filter (Falcon) to obtain single cell suspension, as described above, and plated on Rainbow™ (Biolog) agar plates to evaluate *S. typhimurium* translocation dissemination after overnight culture.

For the *in vivo* experiments with LP-PBF-treated *S. typhimurium* (LP-PBF-treated Sal), 10^9^ CFU of *S. typhimurium* strain SL1344 was pretreated with 5 mg/ml of LP-PBF for 1.5 h at 4°C, then LP-PBF was washed out and LP-PBF-treated Sal was further used for the infection of mice *via* oral gavage. After 6 h, all the mice were euthanized and colon and liver were processed as described above. Cells isolated from colons were plated on Columbia agar with sheep blood (Oxoid) to evaluate bacterial dissemination after overnight culture.

The comparison between pretreatment or coincubation of *S. typhimurium* with the postbiotic was as follows: LP-PBF-treated Sal: prepared as described above; Concomitant administration: 10^9^ CFU/mice *S. typhimurium* strain SL1344 was orally administered concomitantly with 135 mg/kg of LP-PBF. Processing of colons and plating on agar plates was done as described above.

### Histological and immunofluorescence analysis

Cross sections from the colonic and small intestinal specimens were dissected and fixed in 4% formaldehyde and embedded in paraffin. 5 μm samples sections were stained with hematoxylin and eosin or with Alcian Blue-PAS (Thermo Fisher Scientific).

The intestinal tissue samples were fixed overnight in paraformaldehyde, l-Lysine pH 7.4, and NaIO_4_ (PLP Buffer). They were washed, dehydrated in 20% sucrose overnight, and included in optimal cutting temperature compound (OCT) (Sakura). Eight μm cryosections were rehydrated, blocked with 0.1 M Tris–HCl pH 7.4, 2% FBS, 0.3% Triton X-100 a stained with following antibodies: anti-mouse PV-1 (clone MECA32, BD Pharmingen) and anti-mouse CD34 (clone RAM34, eBioscience). Slices were then incubated with the appropriate fluorophore-conjugated secondary antibody. Before imaging, nuclei were counterstained with DAPI. Confocal microscopy was performed on a Leica TCS SP8 laser confocal scanner mounted on Leica DMI 6000B inverted microscope equipped with motorized stage. Violet (405 nm laser diode), blue (488 nm argon laser), yellow (561 nm laser diode), and red (633 nm laser diode) laser lines have been used for excitation. Image J software was used for the analysis.

### RNA isolation and quantitation of gene expression by real-time PCR

Total RNA was purified from cells using DirectZol RNA mini prep kit (Zymo Research) following manufacturer’s protocol. cDNA synthesis was performed using SuperScript III reverse transcriptase (Invitrogen) and random hexamers. Real-time PCR reactions were carried out using the SYBR Green PCR kit (Applied Biosystems). Primers used are listed in Table 1. Gene expression were normalized using *Gapdh* as housekeeping gene. Results were quantified using the 2^–ΔΔCt^ method.

**Table 1:** List of primers used in this study.

Gene	Forward primer (5′→3′)	Reverse primer (5′→3′)
*Gapdh*	ATCAGCAATGCCTCCTGCAC	TGGCATGGACTGTGGTCATG
*Def-α*	CAGGCTGTGTCTGTCTCCTT	TCCTCTATTGCCAGCGACGAT
*Tgf-*β	GCCTGAGTGGCTGTCTTTTGA	GCTGAATCGAAAGCCCTGTATT

### Bacterial DNA extraction from fecal samples and quality control

DNA from fecal pellets was extracted with DNeasy Power Soil Pro kit (Qiagen) following manufacturer’s protocol. DNA quality control was performed with the Agilent 4,200 Tape Station system using the Genomic DNA ScreenTape analysis kit (Agilent, Santa Clara, CA, United States), only DNAs having a DIN > 6.5 were used for library preparation.

### Analysis of the microbiota composition by 16S rRNA gene sequencing

Sample library preparation for Next-Generation Sequencing was performed using the QIAseq 16S/ITS Region Panels kit (QIAGEN), targeting the V3V4 hypervariable regions of the bacterial 16S rRNA gene.

Libraries were checked through TapeStation 4,200 (Agilent Technologies) and quantified using MicroPlate Reader GloMax (Promega). The libraries were then pooled at equimolar concentrations and sequenced on a MiSeq Illumina sequencer; at least 100.000 paired end reads with a length of 275 base pairs (bp) were produced per sample. Quality filtering and adapter trimming of sequencing reads was executed with Trimmomatic v0.39 using the following parameters: ILLUMINACLIP:TruSeq_PE.fa:2:30:7 MINLEN:250 AVGQUAL:30. Sequences of amplification primers and reads with unknown nucleotides (N) were removed using Cutadapt v3.7. High-quality and cleaned sequences were analyzed using the Qiime2 platform (v2022.2). Amplicon Sequence Variants (ASVs) were denoised with the Qiime dada2 denoise-paired command setting the following parameters: --p-trunc-len-f 240 --p-trunc-len-r 240. Q2-feature-classifier, trained on the SILVA138 99% OTUs, specifically on the V3V4 region, was used to perform taxonomic classification. Mitochondria and chloroplast sequences were removed and all the ASVs classified at least at phylum level were retained for the subsequent analysis. Diversity measures (*α*- and *β*-diversity indices) were calculated using the Qiime diversity core-metrics-phylogenetic function with a sampling depth of 50,000 sequences. Alpha diversity was evaluated by Chao1 and Shannon index and represented by box-and-whisker plot. Differences of *α*-diversity indices between experimental groups were evaluated with Welch two sample *t*-test. Raw counts classified at genus-level were normalized using DESeq2 R package and comparisons between experimental groups were conducted using Wald test.

### Biofilm formation in microfluidic devices

Microfluidic channels were fabricated using soft lithography and rapid prototyping. Master molds were fabricated by patterning the negative photoresist SU-8 (MicroChem) on silicon wafers. Positive replicas of the microfluidic channels were obtained by pouring polydimethylsiloxane (PDMS, Sylgard 184, Dow Corning) and agent curing 10:1 (w/w) on the master and degassed in a vacuum chamber to remove bubbles. The cured PDMS was peeled off and connecting holes (inlets and outlets) were created using a biopsy puncher (1.5 mm). The PDMS channels were irreversibly bonded to a glass slide upon treatment with oxygen plasma. The devices were sterilized by UV irradiation before each experiment.

*S. typhimurium* strain SL1344 suspension at optical density OD_600_ of 0.2 was injected into rectangular microchannels (H = 52 μm, W = 400 μm), followed by a period (30 min) of rest in which the bacteria had time to adhere to the inner surfaces of the channels. LP-PBF and surfactants solutions in fresh culture medium were flowed into the channels with syringes at 2 μl/min over the course of 4 h. A fully automated image acquisition routine recorded the position of bacteria on the surface for several hours at different locations along the same channel, for each of the channels (Time lapse = 2 min). Biofilm surface coverage was calculated using MATLAB® where the phase contrast acquisitions were converted into binary images and covered areas were calculated.

### Biosurfactants

LP-PBF was used for biosurfactant purification. Concentrated HCl was added to LP-PBF to bring the final pH to 2.0 and kept overnight at 4°C to precipitate the lipids and proteins. Resulted gray-white precipitates were collected by centrifugation at 10,000 rpm for 20 min at 4°C. For further extraction of biosurfactant compounds, 10 ml of chloroform methanol (2:1 v/v) was added to the pellet and incubated in a rotatory shaker at 30°C for 15 min with 250 rpm agitation. The content was centrifuged at 10,000 rpm for 20 min under cooling condition, the supernatant was collected, while the pellet was resuspended in PBS, considering this a non-organic fraction of biosurfactants, and stored at 4°C. The collected supernatant was evaporated by air drying and the resulting pellet was resuspended in DMSO, considering this the organic fraction of biosurfactants. Analytical quantification of surfactants was performed by Chemservice s.r.l. (Milan, Italy). Whole LP-PBF contains 250–450 mg/kg of total biosurfactants, while the organic fraction contains 29-71 mg/l and non-organic 5–14 mg/l.

Rhamnolipid biosurfactants are glycolipids containing L-rhamnose and ß-hydroxyl fatty acids, with amphiphilic properties (both hydrophilic and hydrophobic). These rhamnolipids, both mono- (R95-Md) and di-rhamnolipids (R95-Dd) product, have been purified from *Pseudomonas aeruginosa* and contain a mixture of rhamnolipids with varying tail length fatty acids (AGEA Technologies).

## Results

### LP-PBF postbiotic treatment can preserve IEB properties *in vitro*

**Figure 1:**
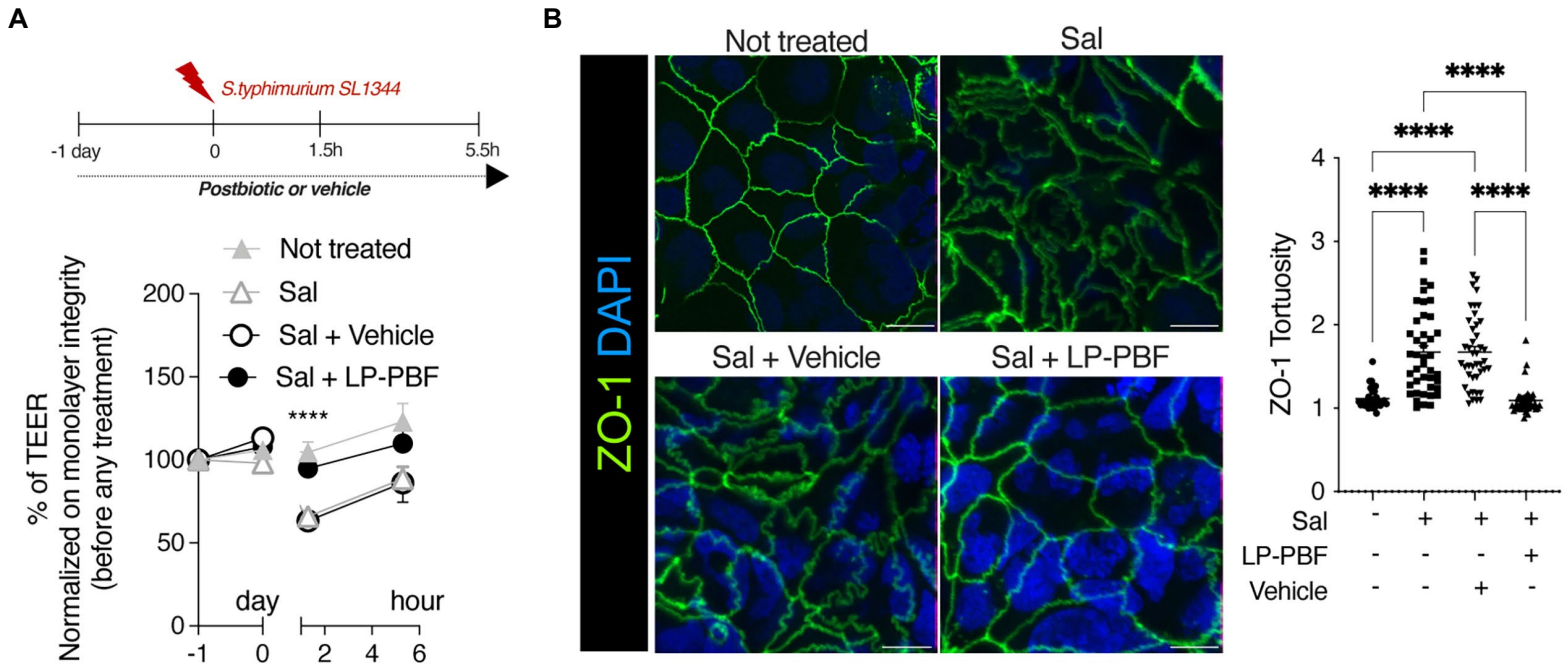
LP-PBF postbiotic treatment is able to preserve IEB properties. **(A)** Graph represents TEER values of Caco-2 cells that were pre-treated overnight with LP-PBF, then infected with *S. typhimurium* SL1344 for 1.5h, followed by recovery phase of 4h in the presence of LP-PBF. * Two-way ANOVA, Tukey’s multiple comparisons test. Above schematic representation of the experimental design. **(B)** Representative immunofluorescence images of ZO-1 done on Caco-2 cells after 1.5h of *S. typhimurium* SL1344 infection. Graph on the right represents quantification of ZO-1 junction tortuosity - ratio between junction length and Euclidean distance between its end. Scale bar - 10mm. Experimental design as in A.* Statistical analysis was evaluated using One-way ANOVA, Tukey’s multiple comparisons test. * p-value < 0.05; ** *p*-value < 0.01; *** *p*-value < 0.001; **** *p*-value < 0.0001.

The primary function of the IEB is to prevent microbiota translocation and preserve immune homeostasis. This is done by regulating gut permeability and allowing only certain molecules to enter or exit the intestine. To assess the role of LP-PBF in preventing colonic epithelium injury *in vitro*, we treated Caco-2 cells overnight with LP-PBF or its vehicle, followed by infection with *S. typhimurium* strain SL1344. TEER values (Srinivasan et al., 2015) were measured at baseline, before and after *S. typhimurium* infection, and after a 4-h recovery phase (removal of *S. typhimurium*). As seen in Figure 1A, infection with *S. typhimurium* induced significant reduction in TEER values in the Caco-2 cell monolayer either alone or in the presence of vehicle. By contrast, pretreatment followed by continuous treatment of Caco-2 monolayer with LP-PBF almost completely abolished the IEB damage caused by *S. typhimurium* infection as represented by unaltered TEER values, similar to those of the non-infected Caco-2 monolayer (Figure 1A).

As TJs are the primary structure that regulate paracellular movement of water and solutes, we set to determine if the combination of postbiotic pretreatment and *S. typhimurium* infection might impact TJ structure, such as altered appearance of cell-to-cell junctions. To assess structural changes in TJs, we analyzed junction tortuosity of ZO-1 by immunofluorescence, calculated as a ratio between junction length and Euclidean distance between its ends as previously published (Thaiss et al., 2018). We observed that LP-PBF alone did not induce changes in ZO-1 tortuosity (Supplementary Figure S2A), while *S. typhimurium* infection significantly increased junctional tortuosity and altered the appearance of cell-to-cell junctions (Figure 1B). By contrast, postbiotic but not vehicle pretreatment protected the TJ structures, avoiding ZO-1 increased tortuosity induced by *S. typhimurium* (Figure 1B). To exclude the possibility that the observed effect of LP-PBF was due to a direct antibacterial effect exerted on *S. typhimurium*, we performed an agar disc diffusion assay and a growth curve of *S. typhimurium* in the presence of postbiotic or vehicle (Supplementary Figures S2B,C). The bacterial growth in all experimental groups was comparable, suggesting that the LP-PBF does not have an antibacterial effect. These results in fact show that LP-PBF has preventive effect on IEB disruption caused by *S. typhimurium* infection.

### LP-PBF postbiotic protects the epithelium from *Salmonella typhimurium* infection *in vivo*

**Figure 2:**
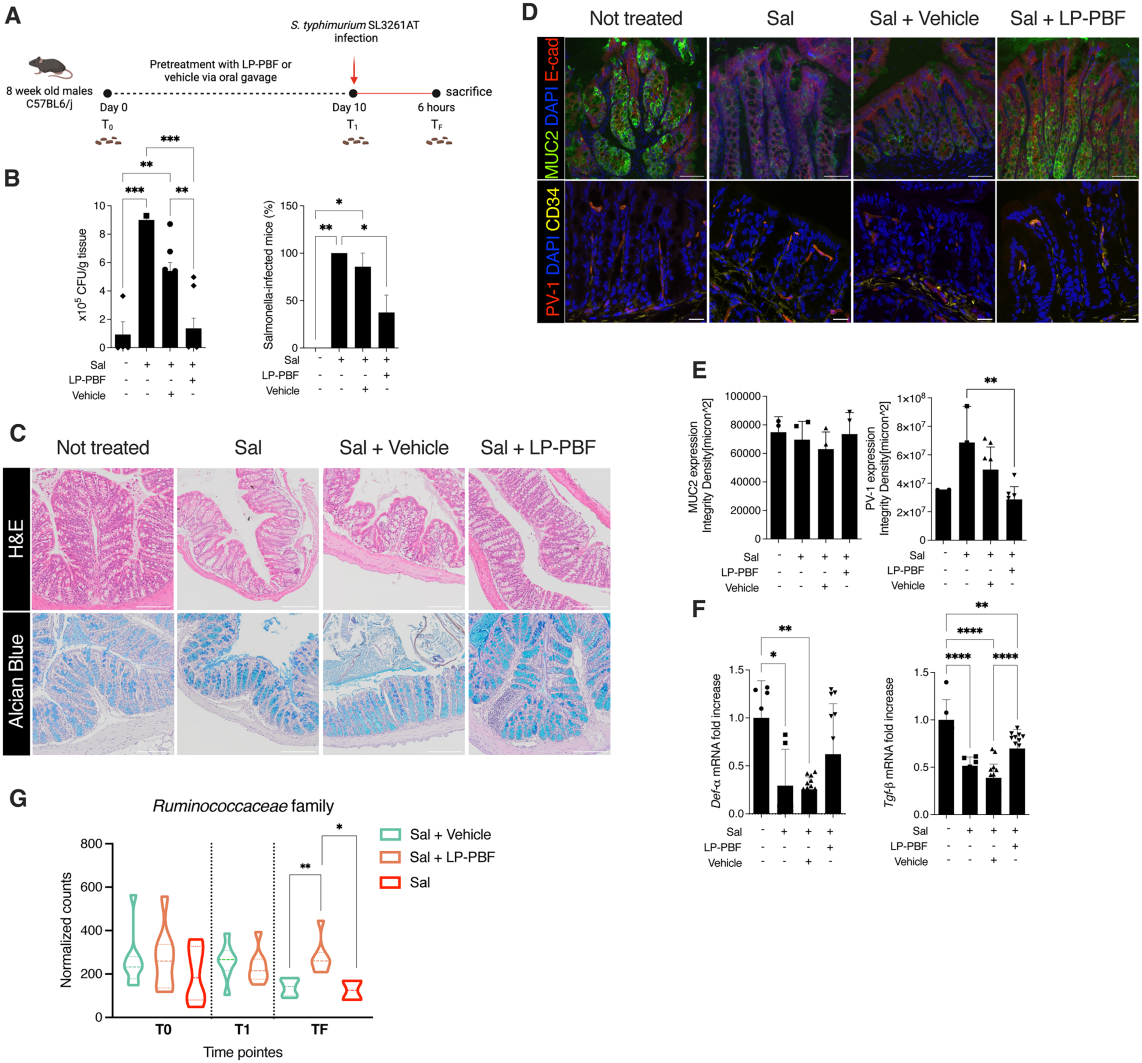
*Lactobacillus paracasei* CNCM I-5220-derived postbiotic prevents the disruption of IEB upon *S. typhimurium* infection. **(A)** Schematic representation of experimental design (created with Biorender). 8-week-old C57BL6/J male mice were treated for 10 consecutive days with 135 mg/kg/day of LP-PBF or vehicle via oral gavage. After the pretreatment, mice were infected with 10^9^ CFU of *S. typhimurium* SL3261AT and upon 6 h were sacrificed. **(B)** *S. typhimurium* SL3261AT dissemination in colon (left) and liver (right) after 6 h of infection. * Statistical analysis was evaluated using One-way ANOVA, Tukey’s multiple comparisons test. **(C)** Panel represent histology analysis performed on FFPE sections: H&E staining (upper panel) and Alcian Blue (bottom panel). Scale bar – 20 μm. **(D)** Representative immunofluorescence analysis performed on colon OCT sections, in following order: MUC2 (upper) and PV-1 (bottom). Scale bar—20 μm. **(E)** Fluorescent intensity quantification of MUC2 (left) and PV-1 (right). * Statistical analysis was evaluated using One-way ANOVA, Tukey’s multiple comparisons test. **(F)** Graph represent fold change of mRNA expression of *α-Defensin* and *Tgf-β* in colon of different groups analyzed. * Statistical analysis was evaluated using One-way ANOVA, Tukey’s multiple comparisons test. **(G)** Graph represent the normalized counts of *Ruminococcaceae* family in different experimental groups and different time points. * Statistical analysis was evaluated using unpaired student t-test. *p-value < 0.05; **p-value < 0.01; ***p-value < 0.001; ****p-value < 0.0001.

Given that LP-PBF has demonstrated to exert a beneficial effect on the IEB function *in vitro*, we next investigated its role in preventing intestinal barrier dysfunction *in vivo*. To do so, we pretreated mice for 10 days with LP-PBF or vehicle via oral gavage prior to challenging mice with *S. typhimurium* strain SL3261AT (Figure 2A), a strain which maintains its invasiveness, but has a reduced ability to survive intracellularly. After 6 h of infection, mice were euthanized and bacterial translocation was assessed in both colon and liver. We observed a significant increase in bacterial colonies in the colons and livers of mice infected with *S. typhimurium* with or without vehicle (Figure 2B). Conversely, we saw that pretreatment with LP-PBF prevented *S. typhimurium* dissemination in the colon and the liver (Figure 2B). However, upon infection with this attenuated strain of *S. typhimurium*, no major changes were evident in histological parameters in the colon, in mucus composition (Figure 2C), or in the level of the secretory protein mucin 2 (Figures 2D,E left graph MUC2), the primary component of the protective mucous layer in the colon (Willemsen et al., 2003). In order for *S. typhimurium* to disseminate into the liver, the gut vascular barrier (GVB) has to be compromised (Spadoni et al., 2015) and this is assessed via the increased detection of plasmalemma vesicle-associated protein-1 (PV-1), a marker of endothelial cell permeability (Liebner et al., 2008; Wang et al., 2012; Spadoni et al., 2015). To investigate the status of the GVB in colon vessels, we performed immunofluorescence staining of PV-1 (Figures 2D,E right panel). PV-1 expression was statistically significantly lower in the group of mice pretreated with the postbiotic, suggesting that LP-PBF has a significant effect on preserving the GVB. Finally, we evaluated the mRNA expression levels of both *α*-Defensin, which represents one of the antimicrobial peptides released by the host, and Tgf-ß, as a marker of an anti-inflammatory response. After *S. typhimurium* infection, we observed a significant reduction of the expression of both genes, which were preserved with pretreatment with LP-PBF (Figure 2F).

**Figure 3:**
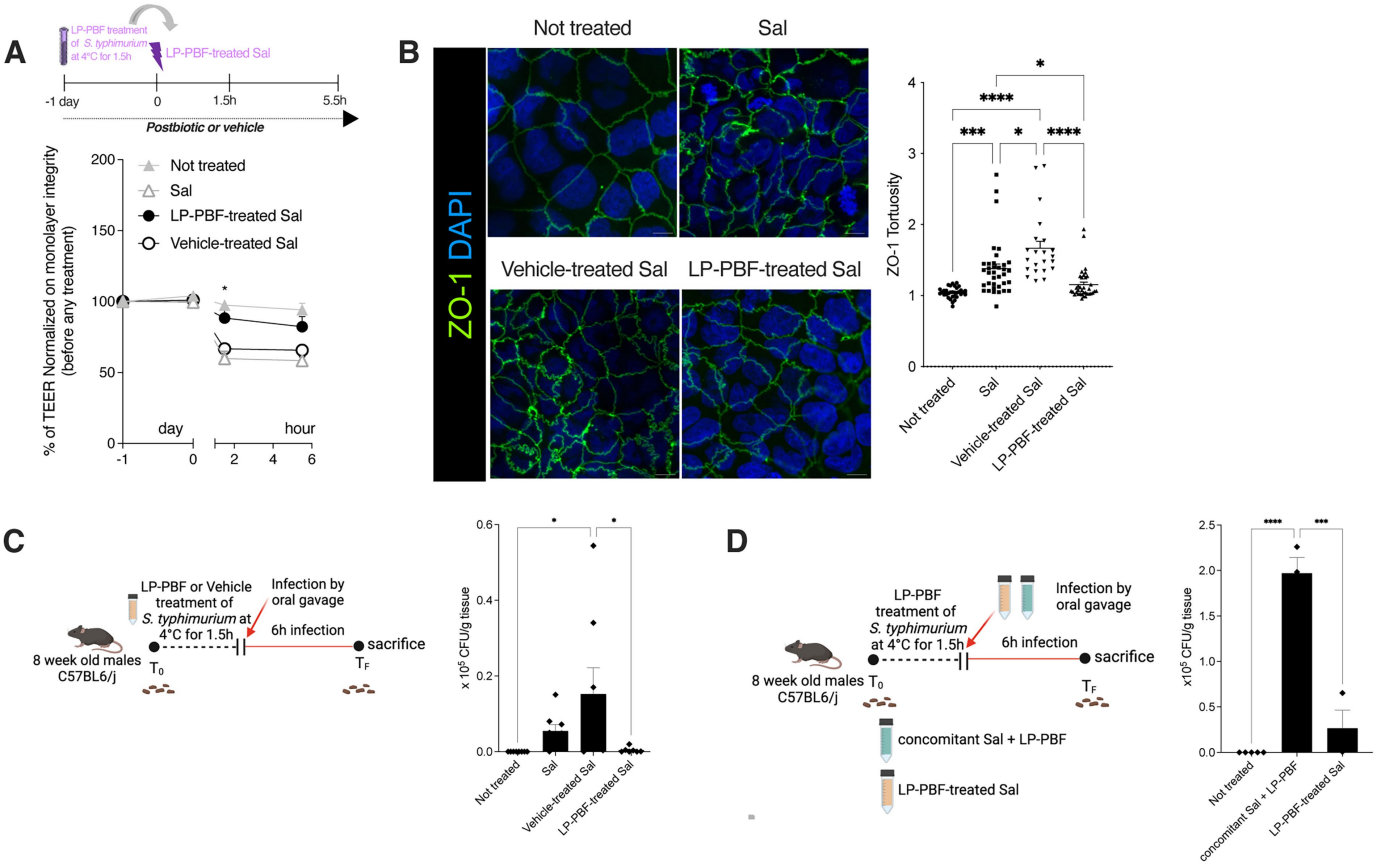
*Salmonella typhimurium* treated with LP-PBF is less invasive and not able to disrupt IEB. **(A)** TEER values of Caco-2 cells pre-treated overnight with LP-PBF, followed by infection with LP-PBF-treated *S. typhimurium* SL1344 (LP-PBF-treated Sal) or vehicle-treated *S. typhimurium* SL1344 (Vehicle-treated Sal). Infected Caco-2 cells were left for 4 h in recovery phase in the presence of postbiotic or vehicle. * Statistical analysis was evaluated using Two-way ANOVA, Tukey’s multiple comparisons test. Schematic representation of the experimental design is present above the graph. **(B)** Representative immunofluorescence images of ZO-1 done on Caco-2 cells after 1.5 h of LP-PBF-treated *S. typhimurium* infection. Graph on the right represents quantification of ZO-1 junction tortuosity—ratio between junction length and Euclidean distance between its ends. Experimental design as in A. * Statistical analysis was evaluated using One-way ANOVA, Tukey’s multiple comparisons test. **(C)** LP-PBF or vehicle-treated Sal dissemination in colon after 6 h of infection. On the left schematic representation of experimental design (created with Biorender). *S. typhimurium* SL1344 was preincubated with either postbiotic or vehicle for 1.5 h at 4°C. Following, 8-week-old C57BL6/J male mice were infected with 10^9^ CFU of LP-PBF-treated or vehicle-treated Sal via oral gavage and upon 6 h were sacrificed. Fecal pellets were collected before and after infection. * Statistical analysis was evaluated using One-way ANOVA, Tukey’s multiple comparisons test. **(D)** Graph represent dissemination of *S. typhimurium* in colon after 6 h of infection with *S. typhimurium* SL1344 in the presence of 135 mg/kg of LP-PBF (concomitant Sal + LP-PBF) and LP-PBF-treated Sal. On the left schematic representation of experimental design (created with Biorender). *S. typhimurium* SL1344 was preincubated with postbiotic for 1.5 h at 4°C. Following, 8-week-old C57BL6/J male mice were infected with either 10^9^ CFU of LP-PBF-treated or S. typhimurium with concomitant presence of one dose of 135 mg/kg LP-PBF and upon 6 h were sacrificed. Fecal pellets were collected before and after infection. * Statistical analysis was evaluated using One-way ANOVA, Tukey’s multiple comparisons test. *p-value < 0.05; **p-value < 0.01; ***p-value < 0.001; ****p-value < 0.0001.

Next, we performed 16S rRNA sequencing analysis to understand the effect of LP-PBF on shaping intestinal microbiota composition and after *S. typhimurium* infection. We analyzed fecal pellets collected at baseline (T0), after pretreatment with postbiotic or vehicle alone (T1), and upon *S. typhimurium* infection (TF). Relative abundance of different phyla and genera present in the two experimental groups (postbiotic and vehicle control) was comparable at baseline and after pretreatment, suggesting that LP-PBF is not inducing major changes in the composition of the intestinal microbiota, as somehow expected as it does not exert antibiotic activity (Supplementary Figures S3A,B). We did not observe major changes also after 6 h of *S. typhimurium* infection in both experimental groups, probably due to the short period of analysis after *S. typhimurium* treatment (Supplementary Figures S3A,B). These results were confirmed when α-diversity Chao and Shannon index were calculated, as there were no evident changes in overall richness or diversity of the microbiota (Supplementary Figures S3C,D). However, we observed a statistically significant difference in the *Ruminococcaceae* family (Figure 2G), whereby LP-PBF prevented the decrease of *Ruminococcaceae* family caused by *S. typhimurium* infection.

Taken together, the above results strongly suggest that LP-PBF postbiotic can protect the intestinal IEB and GVB barriers, thus preventing bacterial translocation in the colon and its dissemination to the liver. Moreover, LP-PBF does not induce gross changes at the level of intestinal microbiota, but it can preserve the abundance of *Ruminococcaceae* family.

### LP-PBF postbiotic neutralizes *S. typhimurium in vitro* and *in vivo*

To evaluate if LP-PBF postbiotic can neutralize *S. typhimurium*, we pretreated *S. typhimurium* SL1344 with the postbiotic or vehicle (LP-PBF-treated Sal or Vehicle-treated Sal) for 1.5 h. Pretreated bacteria were used to infect Caco-2 cells *in vitro* and the TEER was measured (Figure 3A). We observed that LP-PBF-treated Sal did not compromise the integrity of the epithelial monolayer and the TEER values remained similar to those of non-treated monolayers. Conversely, the values of TEER of Caco-2 cells infected with vehicle-treated Sal or untreated Sal dropped similarly. The same was true when we analyzed ZO-1 expression, where LP-PBF-treated Sal prevented the misshaping of cell-to-cell TJs observed after infecting with Vehicle-treated Sal or untreated Sal, thus preserving the low tortuosity of the untreated Caco-2 cells (Figure 3B). To exclude the possibility that postbiotic pretreatment of *S. typhimurium* affected its growth, we performed a growth curve of *S. typhimurium* (Supplementary Figure S4) and confirmed that LP-PBF-treated Sal had the same growth rate as control *S. typhimurium*. These results suggest that LP-PBF treatment of *S. typhimurium* can impede *S. typhimurium* in disrupting the TJ structures *in vitro*, without affecting its growth.

To validate these results *in vivo,* we performed infection of mice with LP-PBF-or vehicle-treated Sal and euthanized them after 6 h to evaluate bacterial dissemination (Figure 3C). We observed that there is a significant decrease of bacterial colonies in the colon of mice infected with LP-PBF-treated Sal, which is in line with the results obtained in our *in vitro* experiments. Of note, *S. typhimurium* was much less invasive when pretreated rather than concomitantly treated with the postbiotic (Figure 3D), suggesting that LP-PBF postbiotic can neutralize *S. typhimurium* to make it less invasive.

These results suggest that LP-PBF postbiotic can exert a beneficial effect on epithelial cells by preserving their barrier properties, but also directly on *S. typhimurium* by making it less invasive, thus less harmful.

### LP-PBF postbiotic exert antibiofilm properties, partly through biosurfactants

**Figure 4:**
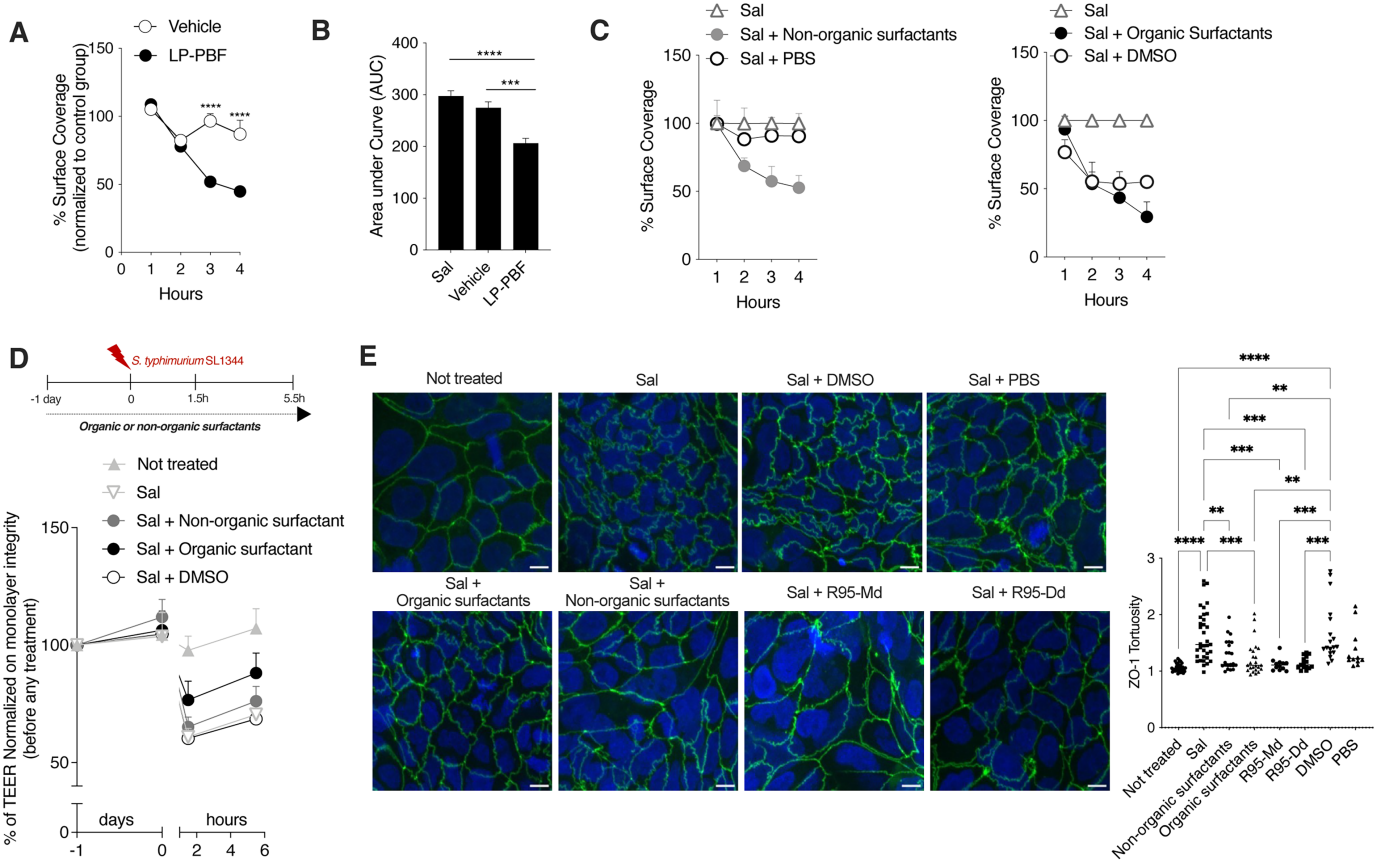
Whole LP-PBF postbiotic and its extracted biosurfactants prevent the formation of *S. typhimurium* biofilm. **(A)** Graph represent quantification of surface coverage during *S. typhimurium* SL1344 biofilm formation in two different experimental groups in the presence of whole LP-PBF postbiotic or vehicle. Values represented are normalized to the control *S. typhimurium* group: vehicle and postbiotic treatment during the time course of 4 h. * Statistical analysis was evaluated using Two-way ANOVA, Tukey’s multiple comparisons test. **(B)** Quantification of area under the curve (AuC) calculated on the graph **(A)**. * Statistical analysis was evaluated using Two-way ANOVA, Tukey’s multiple comparisons test. **(C)** Graph represent quantification of surface coverage during *S. typhimurium* SL1344 biofilm formation in two different experimental groups in the presence of surfactants extracted from LP-PBF. Values represented are normalized to the control *S. typhimurium* group: Organic surfactants and DMSO control (left) and non-organic surfactants and PBS (right) treatment during the time course of 4 h. * Statistical analysis was evaluated using One-way ANOVA, Tukey’s multiple comparisons test. **(D)** TEER values of Caco-2 cells pretreated overnight with organic/non-organic biosurfactants/DMSO, followed by infection with *S. typhimurium* SL1344. Infected Caco-2 cells were left for 4 h in recovery phase with the presence of biosurfactants or the control. Above schematic representation of the experimental design. **(E)** Representative immunofluorescence images of ZO-1 done on Caco-2 cells after 1.5 h of *S. typhimurium* SL1344 infection together with controls (DMSO and PBS), organic or non-organic surfactants, and commercially available rhamnolipids: mono-rhamnolipids (R95-Md) or di-rhamnolipids (R95-Dd). Graph on the right represents quantification of ZO-1 junction tortuosity—ratio between junction length and Euclidean distance between its ends. * Statistical analysis was evaluated using One-way ANOVA, Tukey’s multiple comparisons test. *p-value < 0.05; **p-value < 0.01; ***p-value < 0.001; ****p-value < 0.0001.

The biofilm provides a physical barrier that protects bacteria from adverse environmental conditions. Biofilm-forming microorganisms show resistance to the presence of antibiotics, heating, anaerobic conditions, and varying pH (Mirghani et al., 2022). It has been shown that *L. paracasei* can interfere with biofilm formation of some bacterial pathogens, such as *Streptococcus mutant* and *Streptococcus oralis*, thus interfering with their growth (Ciandrini et al., 2017). Since *L. paracasei* was used to produce the postbiotic used herein, we wanted to address if LP-PBF could prevent *S. typhimurium* biofilm formation. To do so, we followed *S. typhimurium* biofilm formation in a microfluidic device over a course of 4 h and the percentage of surface coverage was calculated for postbiotic- and vehicle-treated channels (Figures 4A,B). Interestingly, the channel where *S. typhimurium* and the postbiotic were both present formed less biofilm when compared to the channel with *S. typhimurium* and vehicle control. These results suggest that LP-PBF exerts an antibiofilm effect similar to that reported for live *L. paracasei*.

Furthermore, it is known that *Lactobacillus* can produce biosurfactants (Rodrigues et al., 2006; Ciandrini et al., 2017) which are surface-active compounds synthesized by a diverse group of microorganisms and are known to hinder biofilm formation (Sambanthamoorthy et al., 2014; Satputea et al., 2016; Mishra et al., 2020). Biosurfactants exist in a wide variety of chemical compounds, such as fatty acids, neutral lipids, phospholipids, glycolipids, and lipopeptides (Cameotra et al., 2010). To elucidate the mechanism through which LP-PBF exerts its antibiofilm effect, we first isolated biosurfactants from LP-PBF and obtained two fractions: organic biosurfactants (soluble in DMSO) and non-organic surfactants (soluble in PBS). We observed that, in this system, only the non-organic fraction could replicate the inhibitory effect of LP-PBF on *S. typhimurium* biofilm formation, while organic surfactants had an effect which was similar to their vehicle, DMSO (Figure 4C). Moreover, we analyzed the effect of these two fractions of biosurfactants on the maintenance of epithelial barrier integrity upon *S. typhimurium* infection as measured by TEER (Figure 4D). Here, only the organic fraction of biosurfactants could partially preserve the epithelial monolayer. Finally, we evaluated the effect of biosurfactants on ZO-1 morphology. In addition to using the LP-PBF, we included commercially available biosurfactants, mono- and di-rhamnolipids that we found to be present in the postbiotic mix, to address if they could recapitulate the same effect. Both organic and non-organic biosurfactants isolated from the LP-PBF, as well as the synthetic rhamnolipids, were able to completely preserve ZO-1 tortuosity at the physiological levels (Figure 4E). Thus, these results suggest that the effect of LP-PBF postbiotic on the preservation of epithelial barrier is partly due to the presence of biosurfactants.

## Discussion

It is well known that probiotics can exert numerous beneficial effects, such as defense against pathogens, metabolism management, immune modulation, and disease prevention (Zhou et al., 2022). Probiotics, however, may also be a threat for immunocompromised individuals or for patients with epithelial barrier disruptions for the risk of systemic translocation. In addition, probiotics need to find the right conditions to exert their beneficial effects that are based on competition with the host microbiota for nutrient supply. Thus, for most of probiotics’ beneficial effects on the host, the viability of bacterial cells that reach the GI tract has to be preserved. Because of these reasons, a new research hotspot has been proposed in the exploitation of postbiotics. Postbiotics are natural molecules released during the normal metabolic activity of live bacteria (Tsilingiri and Rescigno, 2013; Zagato et al., 2020; Aguilar-Toala et al., 2021). What differentiates postbiotics from probiotics is their safety profile as they do not contain bacteria, live or dead, or their fragments. Thus, their functional properties and low toxicity pose postbiotics as a novel approach for re-establishing host-microbe homeostasis, without risking potentially harmful bacterial translocation. Since it is very well known the beneficial effect of different bacterial strains on the host organism (Simeoli et al., 2015; Hossain et al., 2022; Wu et al., 2022), we sought to determine if probiotic-derived metabolites, or postbiotics, could also enhance the well-being of the host. Maintenance of the IEB integrity and function requires a fine-tuned balance among different specialized cells to ensure the physiological and protective crosstalk between intestinal microbes and host immune response. This not only protects the host against invading pathogens and xenobiotic substances, but also aids in nutrient absorption. Restoring IEB physiological functions has been a promising approach to treat chronic inflammatory disease, e.g., IBD, Chron disease, ulcerative colitis (UC) (Catalioto et al., 2011; Fortea et al., 2021).

Likewise probiotics, also postbiotics may differ in their properties according to the strain and the substrate used for fermentation. Indeed, it is well known that postbiotics can have a wide range of activities including modulation of the immune response, anti-inflammatory, anti-proliferative, and anti-cancer effects, among others (Thorakkattu et al., 2022). In this study, we focused on the role of LP-PBF in protecting the integrity of intestinal epithelial barrier using both *in vitro* and *in vivo* systems. We questioned whether LP-PBF could preserve the structure of the IEB even after a strong inflammatory insult such as the infection with an enteric pathogen. In fact, LP-PBF almost completely protected the IEB integrity in an *in vitro* system utilizing the Caco-2 cell line. We showed that the postbiotic could efficiently prevent the disruption of the TJ protein ZO-1 and maintain the cell monolayer barrier properties after challenge with *S. typhimurium* infection. These results were confirmed *in vivo*, where we showed that an extended pretreatment with LP-PBF postbiotic could block bacterial translocation in the gut, and from there to the blood stream. The postbiotic exerted this function by preserving the GVB morphology, as shown by the expression of PV-1, and by maintaining immune and barrier homeostasis *via* preserving the expression of α-Defensin and TGF-β. These findings are important since this type of disruption usually occurs during chronic inflammation. Moreover, upon detailed 16S-rRNA sequencing analysis of microbiota *in vivo*, we found that LP-PBF postbiotic did not have a gross effect on shaping the mouse microbiota; however, the *Ruminococcaceae* family, which is usually altered upon infection or strong antibiotic treatment (Gu et al., 2022), was unaltered upon *S. typhimurium* infection in presence of the postbiotic. *Ruminococcaceae* is a family of strictly anaerobic bacteria, previously correlated with normal, healthy microbiota (De Weirdt and Van de Wiele, 2015). Decreased relative abundance of this genus has been associated with different IBDs, such as UC and Crohn’s disease (Sokol et al., 2008; Joossens et al., 2011; Morgan et al., 2012), and other inflammatory diseases such as hepatic encephalopathy (Bajaj et al., 2012). These findings strongly suggest that LP-PBF postbiotic could effectively preserve the IEB, coupled with a fine-tuning of important microbial family such as *Ruminococcaceae*.

The advantage of the models used was that we could identify two different mechanisms by which LP-PBF is preserving the IEB integrity: (1) on epithelial cells by preserving their structure and barrier properties after challenge with *S. typhimurium*, and (2) on *S. typhimurium* itself, by neutralizing it and rendering it less invasive. Taking all of this into consideration, LP-PBF postbiotic could be used as an effective food supplement to prevent potential pathogen infections and to protect from leaky-gut.

Different microorganisms form biofilms, such as *Escherichia coli*, C*ronobacter sakazakii*, *Staphylococcus aureus*, and *Salmonella* spp. (Hurrell et al., 2009; Patel and Sharma, 2010; Allsopp et al., 2012; Lamas et al., 2018; Liu et al., 2020), a thin layer of microorganisms adhering to the surface of an organic or inorganic structure, together with their secreted extracellular polymeric substances mainly consistent of polysaccharides, secreted proteins and extracellular DNA (Tremblay et al., 2013). Biofilm formation is an adaptable attribute of microbes (Koczan et al., 2011), providing a physical barrier that protects bacteria from adverse environmental conditions. Since it was previously published that different species of *Lactobacillus* genus have an effect on biofilm formation from different bacterial strains (Shokri et al., 2018), we wanted to understand if this anti-biofilm characteristic is partially exerted trough the presence of LP-PBF. Indeed, it almost completely abolished *S. typhimurium* biofilm formation in comparison with the vehicle.

Since it is well known that various species of *Lactobacillus* genus can produce biosurfactants (Gudina et al., 2016; Ciandrini et al., 2017) which are capable of blocking biofilm formation (Morais et al., 2017; Mouafo et al., 2020), we proceeded with extracting biosurfactants from LP-PBF and obtained two different fractions: organic and non-organic surfactants. After testing them in different models, we can confirm that the anti-biofilm ability of LP-PBF is due to a synergistic effect of different postbiotic components. In fact, the non-organic fraction exerted anti-biofilm properties on *S. typhimurium* biofilm formation, while the organic fraction maintained the integrity of epithelial monolayer *in vitro*. Finally, both fractions were efficient in protecting the morphology of TJ structures. Therefore, these results confirm that part of the reported inhibitory effect of *L. paracasei* on biofilm formation is exerted trough different microbial metabolites or molecules present in the postbiotic mix. We can further speculate that the beneficial results of LP-PBF postbiotic are due to a synergistic effect of different components that work at different levels. However, further analysis of the activity of single molecules present in the LP-PBF postbiotic is necessary.

In conclusion, we demonstrate that fermentation of FOS by *L. paracasei* CNCM I-5220 leads to a postbiotic comprising a mixture of different metabolites that showed a preventive effect and maintained the IEB integrity. These molecules are efficient in neutralizing pathogens during an acute phase of the infection, making them a novel approach in preventing different pathological conditions. This study highlights the complexity and beauty of *L. paracasei* CNCM I-5220 postbiotic, showing that its biological properties are due to the combined effect of the different components of the mixture. The alteration of IEB integrity, also referred to as the “leaky gut,” occurs in several pathologies, including the metabolic syndrome, non-alcoholic steatohepatitis, and even cancer and neurodegenerative disorders (Catalioto et al., 2011; Bertocchi et al., 2021; Fortea et al., 2021), suggesting that LP-PBF postbiotic could be applied to help in prevention and management of different pathologies which display an impaired IEB functionality.

## Data availability statement

The data presented in the study are deposited in the NCBI Sequence Read Archive (SRA) repository, accession number ID PRJNA933151.

## Ethics statement

The animal study was reviewed and approved by Italian Ministry of Health (927/2022 and 1054/2015).

## Author contributions

FA and NT designed, performed, and analyzed the experiments. CR and RR designed and performed the experiments on biofilm formation. EN performed the 16S library preparation. DB performed all the bioinformatic analyses. FA, NT, GiP, and MR wrote the manuscript. FA, NT, CR, EN, DB, GrP, RR, GiP, and MR revised the manuscript. GiP and MR conceived and designed the study and analyzed and interpreted the data. All authors contributed to the article and approved the submitted version.

## Funding

This work was supported by Postbiotica S.r.l. We acknowledge support from the Italian Ministry of Health—Fondi 5×1000 Ricerca Sanitaria (to RR).

## Conflict of interest

FA and NT were employed by Postbiotica S.r.l. GiP and MR were founders of Postbiotica S.r.l. MR was chief scientific officer of Postbiotica.

The remaining authors declare that the research was conducted in the absence of any commercial or financial relationships that could be construed as a potential conflict of interest.

## Publisher’s note

All claims expressed in this article are solely those of the authors and do not necessarily represent those of their affiliated organizations, or those of the publisher, the editors and the reviewers. Any product that may be evaluated in this article, or claim that may be made by its manufacturer, is not guaranteed or endorsed by the publisher.

## Supplementary material

SUPPLEMENTARY FIGURE S1 Study workflow. Graphical summary of the study workflow.

SUPPLEMENTARY FIGURE S2 LP-PBF postbiotic does not have anti-bacterial effect on *S. typhimurium*. **(A)** Representative immunofluorescence images of ZO-1 done on Caco-2 cells after overnight treatment with LP-PBF or vehicle. Graph on the right represents quantification of ZO-1 junction tortuosity - ratio between junction length and Euclidean distance between its end. Scale bar - 10mm. Experimental design as in A.* Statistical analysis was evaluated using One-way ANOVA, Tukey’s multiple comparisons test. **(B)** Growth curve of *S. typhimurium* SL1344 (red), *S. typhimurium* SL1344 together with 5mg/ml of vehicle (blue), and *S. typhimurium* SL1344 together with 5mg/ml of LP-PBF (white). Growth curve is represented as optical density (OD600) of bacterial suspension at different time point. **(C)** Result of the antibacterial test using the agar disk diffusion method. a – LP-PBF lot. 19I35 5mg/ml; b – LP-PBF lot. 20I69 5mg/ml; c – Vehicle 5mg/ml; d – Gentamicin 100 ng/ml.

SUPPLEMENTARY FIGURE S3 LP-PBF postbiotic is able to increase *Ruminococcaceae* family upon infection with *S. typhimurium*. **(A)** Bar plots of the phylum-level microbial composition of feces from group of mice treated with *S. typhimurium* in combination with vehicle (Sal + Vehicle) (*n* = 8), and mice treated with *S. typhimurium* in combination with postbiotic (Sal + LP-PBF) (*n* = 8). Relative frequencies are plotted as average of each sample type among the two different experimental groups. **(B)** Bar plots of the genera-level microbial composition of feces from group of mice treated with *S. typhimurium* in combination with vehicle (Sal + Vehicle) (*n* = 8), and mice treated with *S. typhimurium* in combination with postbiotic (Sal + LP-PBF) (*n* = 8). Relative frequencies are plotted as average of each sample type among the two different experimental groups. **(C)** Alpha diversity box plot (Chao1 index) of feces (*n*=8) coming from mice treated with Sal + Vehicle or Sal + LP-PBF. Data are represented using Box and Whisker plots. Box plots display the first quartile, median and third quartile values and the whiskers extend from the hinge no further than 1.5 times the interquartile range. Statistical analysis was evaluated using Welch two sample *t*-test, *p*>0.05. Each data point represents one mouse. **(D)** Alpha diversity box plot (Shannon Entropy) of feces (*n*=8) and LC (*n*=8) coming from mice treated with Sal + Vehicle or Sal + LP-PBF. Data are represented using Box and Whisker plots. Box plots display the first quartile, median and third quartile values and the whiskers extend from the hinge no further than 1.5 times the interquartile range. Statistical analysis was evaluated using Welch two sample *t*-test, *p*>0.05. Each data point represents one mouse.

SUPPLEMENTARY FIGURE S4 LP-PBF does not have anti-bacterial effect on LP-PBF-treated *S. typhimurium*. Growth curve of *S. typhimurium* SL1344 (red), *S. typhimurium* SL1344 pretreated with 5mg/ml of vehicle (blue), and *S. typhimurium* SL1344 pretreated with 5mg/ml of LP-PBF (white). Growth curve is represented as optical density (OD600) of bacterial suspension at different time point.
