# Supplementary figures and images for: Lactobacillus paracasei CNCM I-5220-derived postbiotic protects from the leaky-gut
Source: Front Microbiol. 2023 Mar 20;14:1157164. doi: 10.3389/fmicb.2023.1157164 (PMC10067918; doi:10.3389/fmicb.2023.1157164)

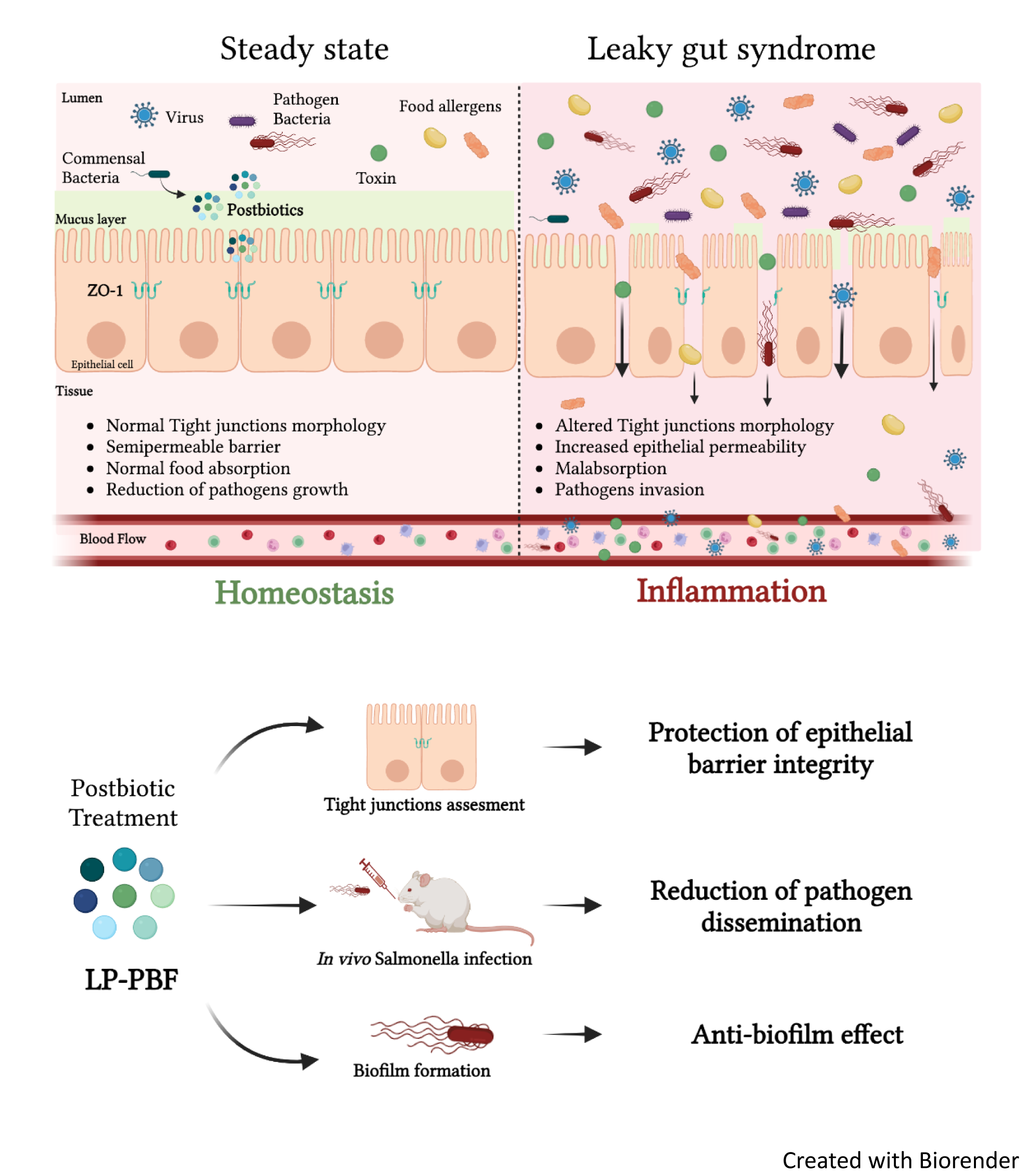

Supplement: SUPPLEMENTARY FIGURE S1 — Study workflow. Graphical summary of the study workflow. [file Image_1.TIF]

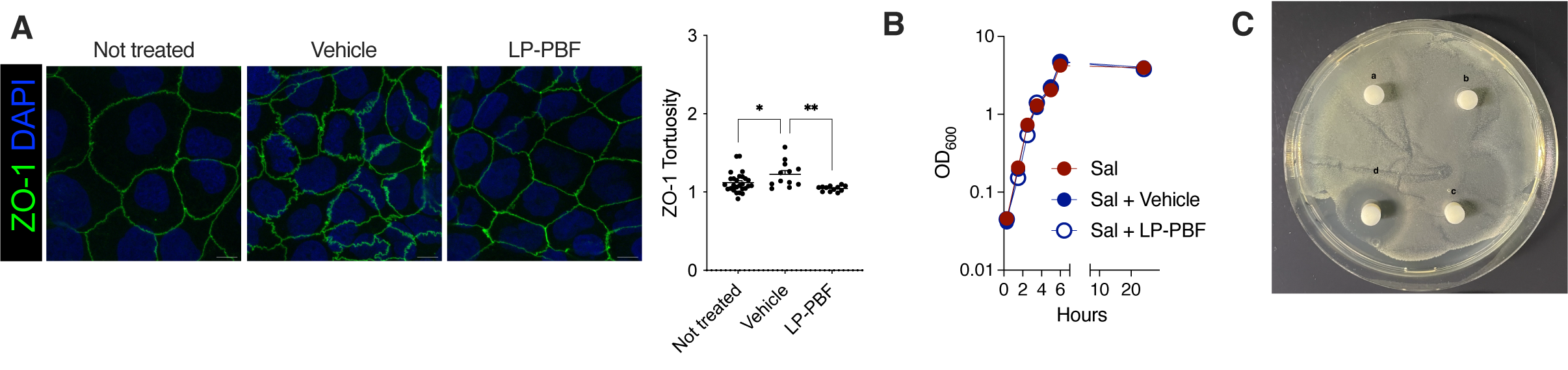

Supplement: SUPPLEMENTARY FIGURE S2 — LP-PBF postbiotic does not have anti-bacterial effect on S. typhimurium. (A) Representative immunofluorescence images of ZO-1 done on Caco-2 cells after overnight treatment with LP-PBF or vehicle. Graph on the right represents quantification of ZO-1 junction tortuosity - ratio between junction length and Euclidean distance between its end. Scale bar - 10mm. Experimental design as in A.* Statistical analysis was evaluated using One-way ANOVA, Tukey’s multiple comparisons test. (B) Growth curve of S. typhimurium SL1344 (red), S. typhimurium SL1344 together with 5mg/ml of vehicle (blue), and S. typhimurium SL1344 together with 5mg/ml of LP-PBF (white). Growth curve is represented as optical density (OD600) of bacterial suspension at different time point. (C) Result of the antibacterial test using the agar disk diffusion method. a – LP-PBF lot. 19I35 5mg/ml; b – LP-PBF lot. 20I69 5mg/ml; c – Vehicle 5mg/ml; d – Gentamicin 100 ng/ml. [file Image_2.TIF]

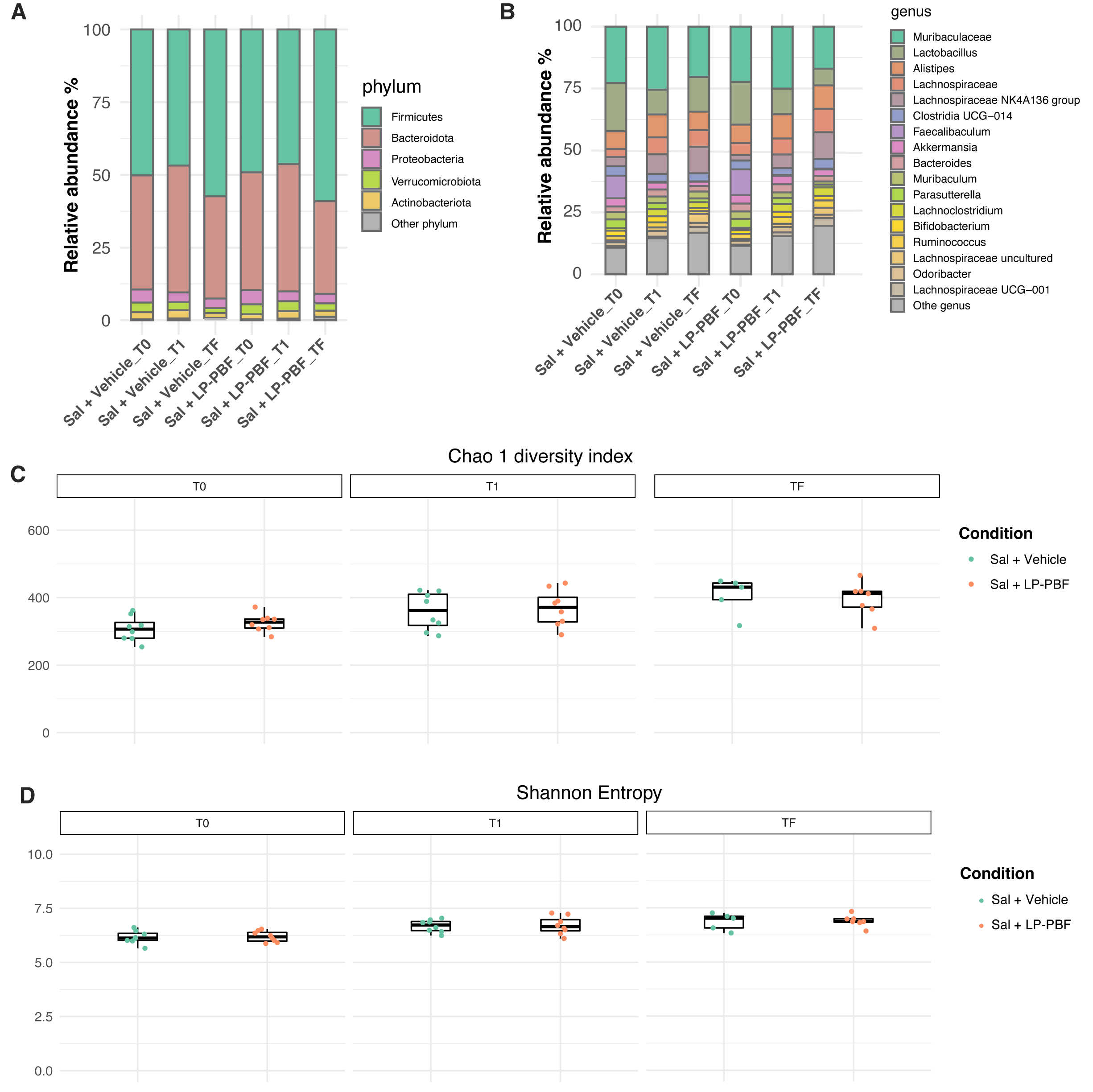

Supplement: SUPPLEMENTARY FIGURE S3 — LP-PBF postbiotic is able to increase Ruminococcaceae family upon infection with S. typhimurium. (A) Bar plots of the phylum-level microbial composition of feces from group of mice treated with S. typhimurium in combination with vehicle (Sal + Vehicle) (n = 8), and mice treated with S. typhimurium in combination with postbiotic (Sal + LP-PBF) (n = 8). Relative frequencies are plotted as average of each sample type among the two different experimental groups. (B) Bar plots of the genera-level microbial composition of feces from group of mice treated with S. typhimurium in combination with vehicle (Sal + Vehicle) (n = 8), and mice treated with S. typhimurium in combination with postbiotic (Sal + LP-PBF) (n = 8). Relative frequencies are plotted as average of each sample type among the two different experimental groups. (C) Alpha diversity box plot (Chao1 index) of feces (n=8) coming from mice treated with Sal + Vehicle or Sal + LP-PBF. Data are represented using Box and Whisker plots. Box plots display the first quartile, median and third quartile values and the whiskers extend from the hinge no further than 1.5 times the interquartile range. Statistical analysis was evaluated using Welch two sample t-test, p>0.05. Each data point represents one mouse. (D) Alpha diversity box plot (Shannon Entropy) of feces (n=8) and LC (n=8) coming from mice treated with Sal + Vehicle or Sal + LP-PBF. Data are represented using Box and Whisker plots. Box plots display the first quartile, median and third quartile values and the whiskers extend from the hinge no further than 1.5 times the interquartile range. Statistical analysis was evaluated using Welch two sample t-test, p>0.05. Each data point represents one mouse. [file Image_3.TIF]

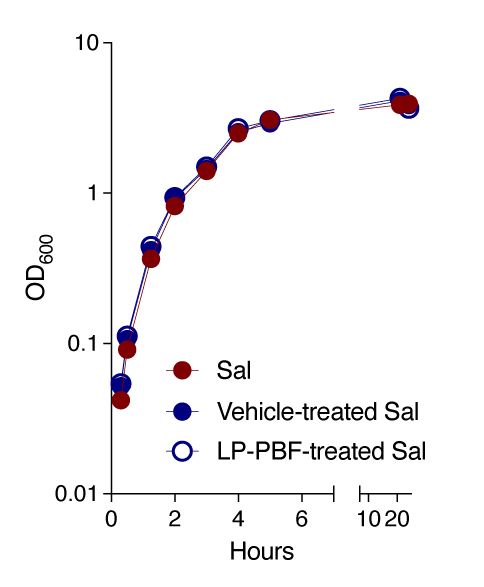

Supplement: SUPPLEMENTARY FIGURE S4 — LP-PBF does not have anti-bacterial effect on LP-PBF-treated S. typhimurium. Growth curve of S. typhimurium SL1344 (red), S. typhimurium SL1344 pretreated with 5mg/ml of vehicle (blue), and S. typhimurium SL1344 pretreated with 5mg/ml of LP-PBF (white). Growth curve is represented as optical density (OD600) of bacterial suspension at different time point. [file Image_4.TIF]
